# Unmarried Hampstead: Much Married Whitechapel

**Published:** 1892-04-09

**Authors:** 


### The Doctor's Armchair.

UNMARRIED HAMPSTEAD: MUCH MARRIED WHITECHAPEL.

It is stated on what may be considered good authority that there are only thirty-six unmarried women of "celibate" age in Whitechapel to every hundred unmarried men of that age. In Hampstead there are three hundred and sixty-six unmarried women to a hundred unmarried men at the same age-period, or ten times the number of possible wives for each unmarried man in Hampstead that are to be found in Whitechapel. From thirty-five to forty-five is here taken as the celibate age-period; that is to say, it is the presumption that those persons of both sexes who do not marry before they reach that period of life will not marry at all.

It is difficult to decide whether Hampstead or Whitechapel is the more to be congratulated. The young women of the North-Western suburb would probably be a little shocked if they found at every dancing party to which they were invited that there were only two young men for every twenty of their own sex. On the other hand, sober parents, who know something of the struggle for existence where human beings are thick on the ground, would much rather their daughters should not marry at all than that they should marry into straitened means' and bring up their children in semi-poverty. The case of Whitechapel, where what is practically universal marriage goes hand-in-hand with universal straitness and deprivation of almost everything that the educated man regards as necessary to life and well-being, acts like a terrifying spectre upon the imagination, and makes one feel that even death itself would be preferable to any sort of marriage which might lead in the Whitechapel direction.

It is often said that the young lady and gentleman of the present period "want to begin where their parents are leaving off"; that they cannot be content with an establishment smaller than the paternal: and this is attributed as a grave fault to the young people. But that is not how the matter strikes the writer. It must be borne in mind that town life is one thing, country life quite another. In the country, that is in the real country, not in a suburban village, a young couple who, as the result of competent training, could make the best use of an orchard, a garden, and a little grass land, might on a couple of hundreds a year be very well off indeed for the first seven years of their lives. They could live in a house quite large enough for vigorous health, they could do without much change of air, and they could surround themselves with all the little accessories and circumstances of refinement which mark off the educated from the uneducated classes. In a word, on such an income they could quite well live in the country without losing the social status so dear to the self-respecting Briton.

But what would two hundred a year do for an educated young couple in Hampstead, or, indeed, in any other wholesome London suburb. It would give them a house so small that, without frequent changes of air, health in it would be impossible; in a street so mean that self-respect would turn sick, and constantly be threatening to die; with a servant so incompetent, food so poor in quality, clean linen so wanting in cleanliness, and everything else requiring money for its purchase so abjectly below the standard of anything like excellence, that the avoidance of physical and mental deterioration would be practically impossible. It is difficult to see how marriage can possibly be thought desirable under circumstances such as these.

There are certain persons of optimistic mind who, having themselves generally found life easy, are apt to think that if anybody wishes for a thing very much he should possess himself of it if he can, and leave the consequences to take care of themselves. That is not a line of conduct which commends itself to science, and certainly not to good morals. Life in a young country, where space is ample and opportunities are numerous, is one thing; but life in an old country, where, practically every inch of space is occupied, and where we are compelled even to build our houses one upon the roof of another, to the height of six or seven storeys, is a thing of another complexion altogether. One of the stiffest of all the problems that the modern political and physiological sciences have to solve in an old civilisation is the reconciliation between the vast crowds of people with their imperious and various wants, and the strictly limited resources which are available for the supply of those wants. The man who has his eyes in his head, and whose thoughts are sober, ought not to be blamed if he is somewhat perplexed in the midst of the millions of our modern England, and if sometimes he even allows himself the luxury of a little dismay.

This very marriage question is only a small part of the practical difficulties that surround statesmen and physiologists in our own country. It is a fact which is not open to question that, as a rule, all men and women ought to marry soon after they have reached adult age. Both the sexes would be healthier and happier for marriage, always provided that other things were equal. The intelligent statesman, and the philosophic physiologist, who see things in the mass as well as in detail, feel it incumbent upon them to aim at such modifications of the hard conditions of modern civilised life as will permit the great majority of men and women to marry, and to marry so as to be advantaged and not injured thereby in the life struggle. To put the matter in brief form: Nature says to all men and women, "Marry." Modern civilisation in crowded Europe says, "Marry by all means, if it so please you; but remember that it is you, and you alone, who must be responsible for the consequences of your marriage."

Is there anything to be gained by presenting to readers these somewhat depressing facts in their native baldness? To which it may be replied: Is there anything to be gained by finding out at the earliest possible date when a patient is suffering from typhoid fever? There is, of course, everything to be gained. When we know the actual truth we can deal with it on right lines. When we are in darkness as to the true nature of the facts, our darkness may induce us to do things that may end fatally. If it be asked, Why does not Hampstead marry? it may be answered, Because Hampstead has the reasonable prudence which education gives. If it be asked why it is that Whitechapel marries so much more than it ought, it is to be replied that if Whitechapel had more science and better morals it would marry much less than it does. The only thing that could restore the just balance of the marriage scale in England, would be more space and a greater number of opportunities for success in life. Where are these to be looked for? Who is the magician to provide us with them?

